# Supplementary material for: Sidedness-Dependent Prognostic Impact of Gene Alterations in Metastatic Colorectal Cancer in the Nationwide Cancer Genome Screening Project in Japan (SCRUM-Japan GI-SCREEN)
Source: Cancers (Basel). 2023 Oct 27;15(21):5172. doi: 10.3390/cancers15215172 (PMC10647889; doi:10.3390/cancers15215172)
Supplement: Supplementary file 1 [file cancers-15-05172-s001.zip › Figure S1.pdf]

(**a**)

Gene	HR	95% CI	*P* value
TP53 (GOF vs. wild-type)	1.47	(1.01 to 2.13)	0.04
TP53 (non-GOF vs. wild-type)	1.13	(0.83 to 1.54)	0.45
KRAS/NRAS (variant vs. wild-type)	1.47	(1.10 to 1.95)	< 0.01
BRAF (V600E vs. wild-type)	1.66	(0.75 to 3.68)	0.21
PIK3CA (variant vs. wild-type)	1.06	(0.70 to 1.59)	0.79
NOTCH1 (sole variant vs. wild-type)	1.21	(0.79 to 1.86)	0.39
NOTCH2 (sole variant vs. wild-type)	1.03	(0.42 to 2.55)	0.94
NOTCH3 (sole variant vs. wild-type)	0.31	(0.15 to 0.63)	< 0.01
FBXW7 (variant vs. wild-type)	1.00	(0.58 to 1.75)	0.99
SMAD4 (variant vs. wild-type)	1.35	(0.72 to 2.51)	0.35
FLT3 (amplification vs. non-amplification)	0.75	(0.51 to 1.11)	0.15
MYC (amplification vs. non-amplification)	1.01	(0.63 to 1.60)	0.98

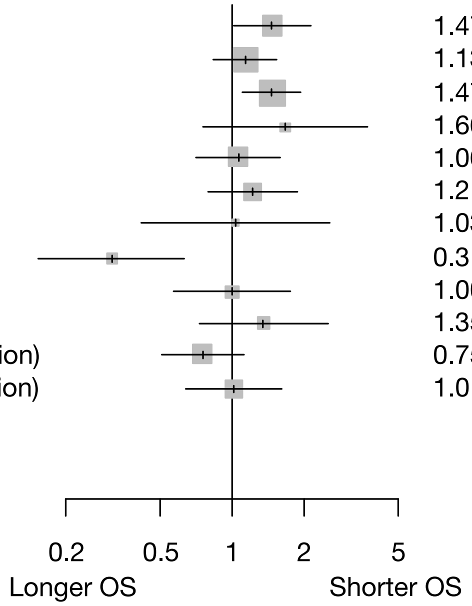

(**b**)

Gene	HR	95% CI	*P* value
TP53 (GOF vs. wild-type)	1.51	(0.90 to 2.54)	0.12
TP53 (non-GOF vs. wild-type)	1.56	(1.01 to 2.41)	< 0.05
KRAS/NRAS (variant vs. wild-type)	1.30	(0.77 to 2.19)	0.32
BRAF (V600E vs. wild-type)	2.47	(1.36 to 4.49)	< 0.01
PIK3CA (variant vs. wild-type)	1.01	(0.61 to 1.67)	0.97
NOTCH1 (sole variant vs. wild-type)	0.80	(0.38 to 1.70)	0.56
NOTCH2 (sole variant vs. wild-type)	1.57	(0.70 to 3.51)	0.28
NOTCH3 (sole variant vs. wild-type)	0.65	(0.33 to 1.31)	0.23
FBXW7 (variant vs. wild-type)	1.23	(0.43 to 3.51)	0.69
SMAD4 (variant vs. wild-type)	1.07	(0.53 to 2.17)	0.85
FLT3 (amplification vs. non-amplification)	1.26	(0.70 to 2.27)	0.43
MYC (amplification vs. non-amplification)	2.34	(1.19 to 4.59)	0.01

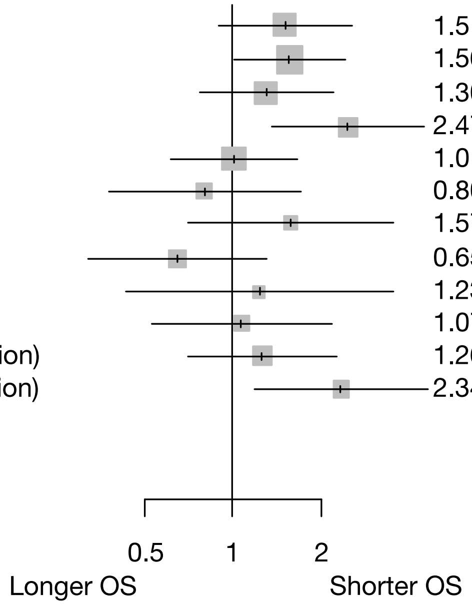

Figure S1. Multivariate analysis including *KRAS/NRAS* instead of *KRAS*. (**a**) Left-sided CRC and (**b**) right-sided CC. CC, colon cancer; CI, confidence interval; CRC, colorectal cancer; GOF, gain-of-function; HR, hazard ratio; Non-GOF, non-gain-of-function; OS, overall survival.
