# Supplementary material for: Sidedness-Dependent Prognostic Impact of Gene Alterations in Metastatic Colorectal Cancer in the Nationwide Cancer Genome Screening Project in Japan (SCRUM-Japan GI-SCREEN)
Source: Cancers (Basel). 2023 Oct 27;15(21):5172. doi: 10.3390/cancers15215172 (PMC10647889; doi:10.3390/cancers15215172)
Supplement: Supplementary file 1 [file cancers-15-05172-s001.zip › Table S1.pdf]

Table S1. Target genes in the Oncomine™ Comprehensive Assay v3.

Hotspot genes				Full-length genes			Copy-number genes		Gene fusions (inter- and intragenic)		
AKT1	ESR1	KIT	PDGFRB	ARID1A	FBXW7	PTEN	AKT1	FGFR4	AKT2	FGFR2	NUTM1
AKT2	EZH2	KNSTRN	PIK3CB	ATM	MLH1	RAD50	AKT2	FLT3	ALK	FGFR3	PDGFRA
AKT3	FGFR1	KRAS	PIK3CA	ATR	MRE11	RAD51	AKT3	IGF1R	AR	FGR	PDGFRB
ALK	FGFR2	MAGOH	PPP2R1A	ATRX	MSH6	RAD51B	ALK	KIT	AXL	FLT3	PIK3CA
AR	FGFR3	MAP2K1	PTPN11	BAP1	MSH2	RAD51C	AXL	KRAS	BRCA1	JAK2	PRKACA
ARAF	FGFR4	MAP2K2	RAC1	BRCA1	NBN	RAD51D	AR	MDM2	BRCA2	KRAS	PRKACB
AXL	FLT3	MAP2K4	RAF1	BRCA2	NF1	RNF43	BRAF	MDM4	BRAF	MDM4	PTEN
BRAF	FOXL2	MAPK1	RET	CDK12	NF2	RB1	CCND1	MET	CDKN2A	MET	PPARG
BTK	GATA2	MAX	RHEB	CDKN1B	NOTCH1	SETD2	CCND2	MYC	EGFR	MYB	RAD51B
CBL	GNA11	MDM4	RHOA	CDKN2A	NOTCH2	SLX4	CCND3	MYCL	ERBB2	MYBL1	RAF1
CCND1	GNAQ	MED12	ROS1	CDKN2B	NOTCH3	SMARCA4	CCNE1	MYCN	ERBB4	NF1	RB1
CDK4	GNAS	MET	SF3B1	CHEK1	PALB2	SMARCB1	CDK2	NTRK1	ERG	NOTCH1	RELA
CDK6	H3F3A	MTOR	SMAD4	CREBBP	PIK3R1	STK11	CDK4	NTRK2	ESR1	NOTCH4	RET
CHEK2	HIST1H3B	MYC	SMO	FANCA	PMS2	TP53	CDK6	NTRK3	ETV1	NRG1	ROS1
CSF1R	HNF1A	MYCN	SPOP	FANCD2	POLE	TSC1	EGFR	PDGFRA	ETV4	NTRK1	RSPO2
CTNNB1	HRAS	MYD88	SRC	FANCI	PTCH1	TSC2	ERBB2	PDGFRB	ETV5	NTRK2	RSPO3
DDR2	IDH1	NFE2L2	STAT3				ESR1	PIK3CB	FGFR1	NTRK3	TERT
EGFR	IDH2	NRAS	TERT				FGF19	PIK3CA			
ERBB2	JAK1	NTRK1	TOP1				FGF3	PPARG			
ERBB3	JAK2	NTRK2	U2AF1				FGFR1	RICTOR			
ERBB4	JAK3	NTRK3	XPO1				FGFR2	TERT			
ERCC2	KDR	PDGFRA					FGFR3				
